# Complete reconstruction of the crystalline lens shape from OCT images acquired with off-axis viewing

**DOI:** 10.21203/rs.3.rs-7957290/v1

**Published:** 2025-12-04

**Authors:** Alvaro de la Peña, Javier Rodriguez, Estela Vadillo, Celia Talaván-González, Ester Carreño, Alberto de Castro, Susana Marcos, Eduardo Martinez-Enriquez

**Affiliations:** 1. Instituto de Óptica “Daza de Valdes”, Madrid, Spain.; 2. Department of Epidemiology of Chronic Diseases, National Center for Epidemiology, Instituto de Salud Carlos III, Madrid, Spain; 3. Ophthalmology Department, La Paz University Hospital, Madrid, Spain.; 4. Flaum Eye Institute, University of Rochester, Rochester, NY, United States.; 5. Center for Visual Science, University of Rochester, Rochester, NY, United States.

**Keywords:** Eye, crystalline lens geometry, aging, presbyopia, cataract, Optical Coherence Tomography

## Abstract

The crystalline lens of the eye is an optical structure that, together with the cornea, focuses images onto the retina. In a young and healthy eye, the lens is transparent and adjust its shape to focus on near and distant objects (accommodation). With age, it loses this flexibility (presbyopia) and may later become opaque (cataract). Understanding the full three-dimensional geometry of the crystalline lens is essential for studying age-related lens growth and accommodation mechanisms, as well as for the selection and design of customized intraocular lenses for implantation in cataract surgery. While Optical Coherence Tomography (OCT) allows imaging of the central lens region visible through the pupil, conventional in vivo measurements cannot capture the full lens shape, particularly at the periphery, due to iris obstruction. In this study, we present a novel method to reconstruct the full three-dimensional shape of the crystalline lens by combining OCT images acquired with light entering at different angles. Using this approach, full lens reconstructions were achieved in eight subjects, and key geometrical parameters - diameter, volume, and surface area- were quantified, showing strong positive correlations with age. Comparisons with estimates derived from pupil-limited regions showed strong agreement, thereby validating these estimation methods.

## 1. Introduction

Quantifying the full shape of the crystalline lens in the human eye plays an important role in numerous clinical and surgical applications. These include cataract surgery planning, such as intraocular lens (IOL) selection or sizing of accommodating IOLs, and the design of next-generation of IOLs ^1^. Furthermore, accurate reconstruction of the lens’s full three-dimensional (3-D) morphology is essential for understanding ocular growth in children, including the role of crystalline lens remodeling in emmetropization, and how its failure can lead to myopia ^2,3^. Accurate lens geometrical models are therefore of interest in the understanding and development of correcting alternatives for myopia and presbyopia. They also provide the geometrical information needed for biomechanical modeling of the lens for studying accommodation ^4^.

Optical Coherence Tomography (OCT) ^5^ is commonly used to capture 3-D images of the eye due to its good trade-off between speed and resolution and its availability in clinical settings. Nevertheless, it provides access only to the limited central portion of the lens visible through the pupil because the iris blocks the incident light, preventing obtaining information of the periphery of the lens. Some imaging modalities have been employed to assess the full geometry of the crystalline lens, including its peripheral regions. Magnetic Resonance Imaging (MRI) and Ultrasound Biomicroscopy (UBM) have demonstrated the capability to visualize the entire lens structure ^6–10^. However, MRI is limited by relatively low spatial resolution (~100 μm), prolonged acquisition times (several minutes), high cost, and limited clinical availability. UBM offers improved resolution (~40 μm) and faster imaging (milliseconds), but requires contact with the ocular surface and a skilled operator, implying patient discomfort and clinical constraints.

In absence of images of the peripheral lens geometry with OCT, the *intersection approach* has been proposed to estimate the lens diameter from the portion of the lens visible through the pupil. This method extrapolates the lens periphery by fitting two parametric surfaces (usually circles) to the visible anterior and posterior lens surfaces and determines the equatorial diameter from their intersection. The intersection approach has been applied in several studies ^11–14^ and is implemented in commercial anterior segment OCT systems, including CASIA2 (Tomey, Nürenberg, Germany) and Catalys femtosecond laser platform (Johnson & Johnson Vision, Santa Ana, CA, USA). However, previous investigations on ex-vivo lenses measured with OCT, in which the full geometry of the lens is visible because the iris is removed, demonstrated that the intersection approach introduces systematic errors, notably overestimating both the equatorial diameter and lens volume (by 15 % and 11 % in average respectively), while underestimating the position of the equatorial plane by shifting it anteriorly (by 17 %) ^15^.

In previous studies, we proposed two methods to accurately estimate the full shape of the crystalline lens from its visible central region ^15,16^. Both methods rely on geometric models trained with ex-vivo OCT data of complete lens geometries. One approach uses parametric descriptions of different regions of the lens ^15^, while the other applies a basis decomposition into *eigenlenses*. In this latter approach, each lens shape is represented as a weighted sum of a small set of *eigenlenses*, with the most significant *eigenlenses* capturing the dominant deformation patterns across a training set of lenses ^16^. These full-shape estimation methods have been applied to study changes in lens geometry with accommodation ^17,18^ and with refractive error ^17,19^. The accuracy of the *eigenlens* representation has been validated in isolated ex vivo lenses ^16^ and in lenses mounted on a lens stretcher that simulate physiological accommodation ^20^. In addition, their ability to capture in vivo shape changes with accommodation has been supported indirectly through comparisons with state-of-the-art MRI measurements ^17^. However, a direct in vivo validation of these methods is still lacking.

In this study, we used off-axis imaging to access the peripheral regions of the crystalline lens. A previous study used oblique OCT scanning with five volumetric image acquisitions to enlarge the visible portion of the posterior crystalline lens surface ^21^, achieving a 17% increase in volumetric lens coverage. However, that work neither imaged the peripheral regions nor reconstructed the true 3-D geometry of the lens. Previous works have proposed methods for stitching multiple 3-D OCT volumes, but they were not developed for in vivo crystalline lens imaging ^22–25^.

The current study presents two key advancements in the imaging and reconstruction of the human crystalline lens using OCT. First, we present, to the best of our knowledge, the first OCT images that capture the full peripheral contour of the crystalline lens. These images extend beyond the central optical zone typically visualized in previous studies with both laboratory-developed and commercial OCT systems, as well as beyond earlier attempts to enlarge the visible portion of the posterior lens surface ^21^. Second, we demonstrate a complete 3-D reconstruction and quantification of the full lens geometry directly from the OCT data, with no reliance on extrapolation as previous estimation methods ^11,12,15–17^. To image the peripheral regions of the crystalline lens, OCT scans were acquired at multiple fixation angles, resulting in off-axis illumination. Measurements from different angles revealed different regions of the posterior lens surface and extended coverage to the equatorial region of the lens. A complete 3-D reconstruction of the lens geometry was then achieved by registering all volumetric datasets within a common coordinate system.

This study reports reconstructions of the full crystalline lens shape in eight eyes and quantifies several geometric parameters, including lens volume, diameter, and surface area. In addition, we compare the proposed method with previously published estimation models^16,17^, based on *eigenlenses*, thereby validating their applicability in vivo.

## 2. Methods

### 2.1 Subjects

Measurements were acquired on eight eyes from eight subjects with no ocular pathology or surgery, with a mean ± standard deviation age of 29 ± 6 y/o (range: 23 to 40 y/o) and a mean refractive error of −1.6 ± 2.9 D (range: −6.75 D to +2 D). Measurements were performed under pharmacologically induced mydriasis, by one drop of tropicamide 10 mg/ml and phenylephrine 100 mg/ml to dilate the pupil. The subjects signed informed consents that had been approved by Institutional Review Boards “Comité de Ética del CSIC” after the nature of the study had been explained, in accordance to the tenets of the Declaration of Helsinki. All participants in this study provided consent for publication.

### 2.2 OCT System

The OCT images were acquired using the commercial OCT system ANTERION (Heidelberg Engineering, Heidelberg, Germany) that uses a swept-source laser with a wavelength of 1300 nm at a speed of 50,000 A-scans/s. The axial resolution is <10 μm in tissue. It includes an integrated infrared (IR) camera and an active eye tracking mechanism to ensure stable and precise image acquisition. The ANTERION system includes four different measurement modalities: Cornea App, Cataract App, Metrics App, and Imaging App. In this work, we used the Imaging App to obtain images with personalized radial scanning consisting of 65 meridional B-scans (meridians) with a 14 mm lateral scan range each and 256 A-scans/B-scan. These parameters were chosen to achieve a good trade-off between high spatial resolution and total acquisition time of a 3-D data set. The images obtained from the Imaging App are raw images in pixels, which must be converted to millimeters using calibration information as explained below. More details on the ANTERION OCT system can be found in ^26^.

### 2.3 Data acquisition

**Figure 1.**
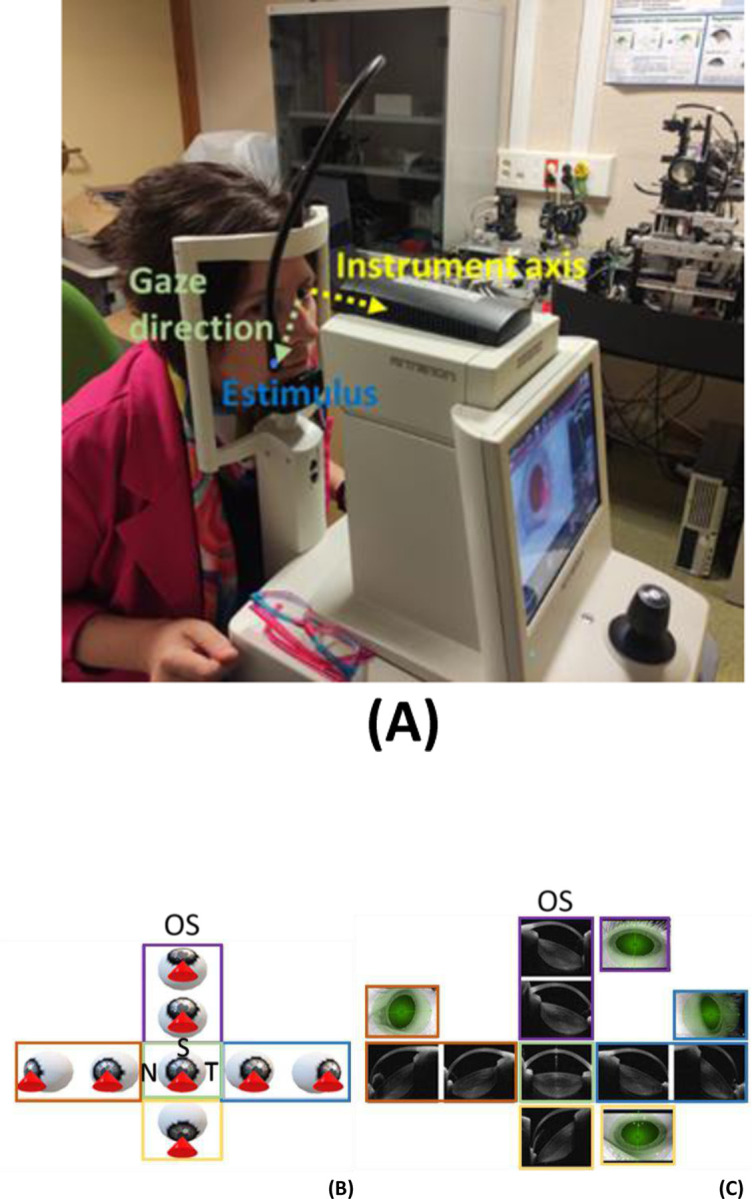
OCT imaging using lateral incidence at 8 different eye orientations. (A) Measurement setup, showing the external stimulus, the gaze direction and the instrument axis. (B). Illustration of the 8 different fixations for OCT acquisition. The orientation of the eye illustrates the eye rotation with fixation. The red cone indicates the beam incidence. (C) Example of a representative OCT meridian (extracted from each volumetric scan) and pupil camera image for each corresponding orientation. For clarity, pupil camera images are presented only at higher lateral incidence angles: 45˚ nasally and temporally, and 30˚ superiorly. N, S and T stand for Nasal, Superior and Temporal, respectively. Colored lines in boxes indicate incidence: Green line: normal incidence; Red: nasal incidence (30 and 45 deg.); Blue: temporal incidence (30 and 45 deg.); Purple: superior incidence (20 and 30 deg.); Yellow: inferior incidence (45 deg.).

OCT images were acquired from one eye (OS in 7 subjects and OD in 1 subject). The eye was imaged with light incident from 8 different orientations (i.e., obtaining 8 volumetric 3-D measurements, one for each orientation), using an external stimulus in order to measure the periphery of the lens from different areas. These orientations were: on axis (OCT beam centered, i.e., typical OCT measurements); subject gazing nasally at, approximately, 30° and 45°; subject gazing temporally at 30° and 45°; subject gazing superiorly at 20° and 30°; and subject gazing inferiorly, at 45°. Figure 1A illustrates the measurement setup with images collected in one of the subjects. Figure 1B presents a schematic of the different orientation angles measured (left), and an example of one of the meridians obtained (extracted from each volumetric scan) and the corresponding pupil camera image for each orientation (right). The complete measurement session for a single eye lasted approximately 20 minutes following pupil dilation, which included the change in fixation target and acquisition of a volumetric set per fixation condition. Images containing artifacts (e.g., motion, eyelid interference) were excluded. To evaluate repeatability, three volumetric datasets were acquired for each fixation condition (i.e., light orientation) in two subjects.

### 2.3 Methodology to obtain the full shape of the crystalline lens

**Figure 2.**
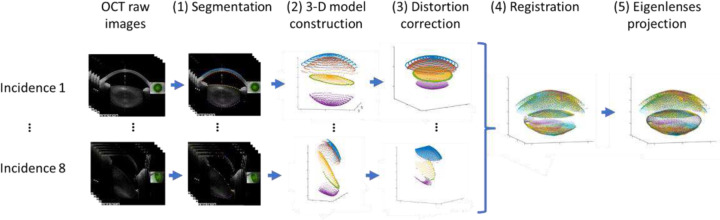
Main process involved in the construction of the full shape of the crystalline lens

Figure 2 illustrates the algorithm developed to obtain the 3-D full shape of the crystalline lens. It includes five steps: (1) OCT images segmentation; (2) 3-D model reconstruction for each incidence angle; (3) distortion correction; (4) registration of the 3-D models from different incidence angles; and (5) *eigenlenses* projection.

#### 2.3.1 OCT images segmentation

The segmentation process involves identifying the anatomical surfaces of interest within the OCT images and classifying them as the anterior corneal surface, posterior corneal surface, anterior lens surface, posterior lens surface, and iris.

For normal and low incidence angles, an algorithm based on previous publications ^18,27^ was developed to automatically segment each OCT image. In this work, specific improvements were introduced to enhance segmentation performance, including: (i) the use of iris segmentation to restrict the processing area to the pupil, thereby improving the accuracy of crystalline lens segmentation; and (ii) the removal of eyelash artifacts in anterior cornea segmentation through an iterative process. In this process, the detected edges were first fitted to a low-order polynomial, and samples located beyond a predefined distance of 3 standard deviations from the fitted polynomial were discarded.

As the automatic algorithms failed at high incidence angles, a manual segmentation approach was developed for those cases. Specifically, for each anatomical surface of interest in every OCT image, at least five points were selected by manually clicking with the mouse along the visible boundary. The manual procedure followed several guidelines: (i) the iris was segmented by selecting the most internal points on the “left” and “right” parts of the pupil (see green points in Supplementary**Error! Reference source not found.**); (ii) to avoid losing information in subsequent processing, points were chosen to cover all visible regions of each surface. Remarkably, if, for example, a large portion of the anterior lens surface was visible but not “covered” by the cornea (i.e., the segmentation domain was lower in the cornea than in the anterior lens), the uncovered region of the lens would be lost during the distortion correction process; (iii) in most cases, the first and last points selected on the anterior lens corresponded to the iris segmentation.

Although time-consuming, this method provided reliable and consistent segmentation results across multiple orientations and subjects. Furthermore, as a surface smoothing is performed in the 3-D model reconstruction process (see next subsection), small imperfections in the manual segmentation are effectively minimized.

All segmentation results were visually inspected, and manual segmentation was repeated whenever the outcome was deemed unsatisfactory. Importantly, segmentation results were consistent across four independent operators, showing no significant differences.

Supplementary Figure S1 shows segmentation examples on specific meridians of 3 different incidence angles for the same subject.

#### 2.3.2 3-D model reconstruction

After segmentation of all the images, the 3-D geometry was reconstructed for each of the eight different incidence angles. The coordinates of the segmented surfaces were first converted from pixels to millimeters (9.2 µm/pixel), using calibration data obtained from measurements of a reference grid and calibration spheres with known radius of curvature. The meridians were then registered into a common 3-D space by mapping the segmented surfaces according to their corresponding scanning angle (from 0 to π, with angular sampling at intervals of 0.048 rad, i.e., 2.7 °) and direction (i.e. where the scan starts and ends, e.g., from the nasal-superior quadrant to the temporal-inferior quadrant). Finally, the ocular surfaces were approximated using Zernike polynomials ^28,29^, providing smooth and uniformly sampled 3-D geometries. Specifically, 15 Zernike terms were used, and the coefficients were found by fitting each surface using least-squares.

**Figure 3.**
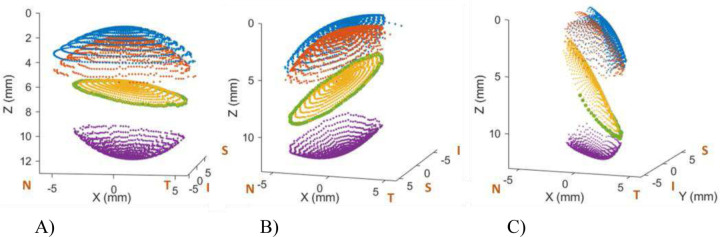
3D model reconstruction (A). Normal incidence; (B) Nasal 30 degrees incidence; (C) Temporal 45 degrees incidence. N, S, T and I stand for Nasal, Superior, Temporal and Inferior respectively. The blue color indicates anterior cornea surface; red, posterior cornea surface; yellow, anterior lens surface; purple, posterior lens surface; and green indicates the iris. Examples are for Subject #1 (OS).

Figure 3 illustrates the volumetric models obtained for three different incidence angles for subject #1 (OS), namely, normal incidence, nasal (30 degrees) incidence and temporal (45 degrees) incidence.

#### 2.3.3 Optical distortion correction

The aim of optical distortion correction is to recover the true geometry of ocular structures by compensating for the geometric distortions introduced by refractive elements in the anterior eye. In particular, when imaging the posterior surface of the lens, light rays are refracted by the cornea and the anterior surface of the lens, altering both their propagation direction and optical path length due to surface curvature and refractive index differences ^30^.

To correct for these effects, the anterior corneal surface data was exported into an optical design and simulation program (Zemax, Ansys, PA). Within the software, rays corresponding to the OCT sampling region were traced, and their intersections with the anterior corneal surface were determined. The refracted direction vectors of these rays were then computed. For reconstruction of the posterior corneal surface, each ray was traced further along its refracted path for a distance equal to the optical path length (measured between the anterior and posterior corneal surfaces in the OCT images), divided by the group refractive index of the cornea. An analogous iterative reconstruction procedure was applied to the crystalline lens. Specifically, the anterior lens surface was reconstructed first, followed by the posterior surface, ensuring that the influence of refraction at each preceding boundary was accounted for in the subsequent step. Supplementary **Error! Reference source not found.**S2 illustrates the optical distortion correction process, showing the uncorrected and corrected surfaces for subject #1. As will be further elaborated in the discussion section, it is essential to perform this correction using three-dimensional ray-tracing algorithms rather than applying independent two-dimensional corrections to each meridian, as is commonly implemented in certain commercial OCT systems. The group refractive indices used in the reconstruction were 1.385 for the cornea ^31^, 1.345 for the aqueous and 1.417 for the crystalline lens ^32,33^.

#### 2.3.4 Registration of 3-D measurements acquired from different incidence angles

The eight 3-D models reconstructed from different incidence angles, each representing a distinct portion of the crystalline lens, were subsequently registered within a common coordinate system to generate the final geometry of the complete lens. To this end, all 3-D models were rotated and translated such that the iris was aligned across all incidence angles. The iris was chosen as the reference structure because it was visible in nearly all meridians across measurements. Specifically, the segmented iris points were approximated by a plane, and the normal vector to this plane was used to correct the tilt by applying a rotational transformation. Subsequently, the iris points were fitted to a circle, and the displacement vector along the X, Y, and Z axes required to place the circle center at the coordinate origin (0,0,0) was calculated. The resulting rotation matrices and displacement vectors were then applied to all reconstructed surfaces.

**Figure 4.**
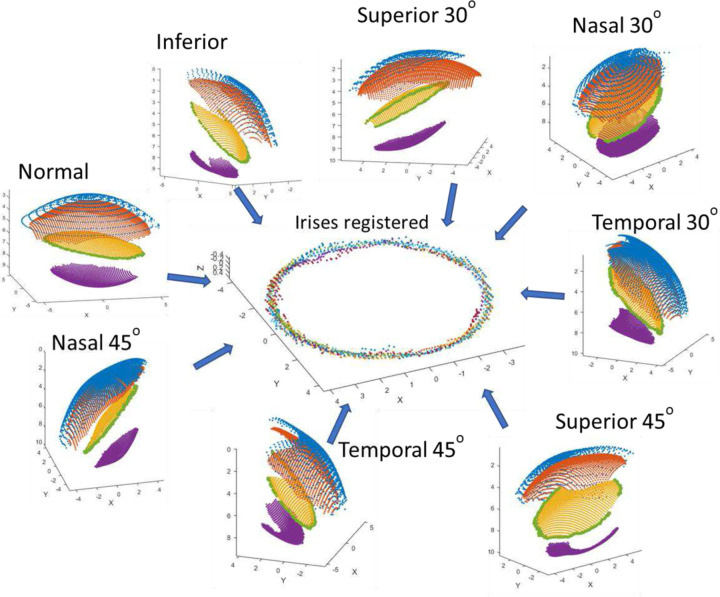
Registration process of the eight 3−D models from the measurements at different incidence angles and their corresponding Iris data after registration. Examples are for Subject #1 (OS).

Figure 4 shows the eight 3-D models from the measurements at different incidence angles and in the center of the figure the eight irises in the same coordinate system (after the registration). Supplementary Video S3 shows how this process is performed dynamically.

**Figure 5.**
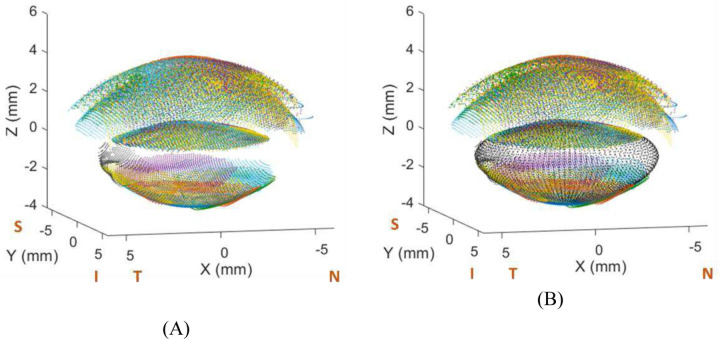
Registered models, fitted to *eigenlens* basis. (A) Combination of data for 8 incidences after the registration process; (B) Data from A, with *eigenlens* fitting superposition (in black). Dark blue: Normal incidence; Red: Inferior incidence; Yellow: Superior 30 deg. incidence; Purple: Superior 45 deg. incidence; Green: Nasal 30 deg. incidence; Blue: Nasal 45 deg. incidence; Olive: Temporal 30 deg. incidence; Charcoal: Temporal 45 deg. incidence. See supplementary video S4 to see the registration process step by step. N, S, T and I stand for Nasal, Superior, Temporal and Inferior respectively.

Following initial registration, the alignment was refined using an iterative closest point (ICP) algorithm ^34^ to optimize the rigid transformation between the eight 3-D models. This algorithm minimized the mean squared error between the point sets by iteratively updating rotation and translation parameters. This step is a refinement to correct the minimum errors that may have occurred in the rotations and translations, especially in the most extreme incidence angles. Figure 5A shows the eight incidences after the registration process, where each color represents the 3-D model of each incidence.

Supplementary video S4 shows the registration of the different incidences dynamically.

#### 2.3.5 Smoothing the full shape crystalline lens using eigenlenses

Finally, the reconstructed and registered 3-D model of the crystalline lens full shape is projected onto a base of *eigenlenses*. This step is very useful for three important reasons: (i) to obtain a compact representation of the lens shape, useful in applications as intraocular lens position and tilt estimation in cataract surgery ^35,36^; (ii) to smooth the reconstructed surface, filtering out noise and local irregularities that may result from imaging artifacts or registration inaccuracies; and importantly (iii) to obtain the lens shape in the full domain, including some areas that were not completely visible in the OCT images (i.e., obtain a closed form shape of the lens). This is further discussed in the Results and Discussion sections. Specifically:

1. The 3-D crystalline lens full-shape dataset were first aligned by centering laterally at the anterior lens maximum elevation point and axially at the midpoint between the anterior and posterior surfaces. The aligned data were then transformed into polar coordinates and interpolated onto a fixed sampling grid consisting of P=100 equidistant elevation angles and Q = 100 equidistant azimuth angles. This grid defines the lens surface with M = P×Q =100 ×100 = 10000 points, from which the surface elevation values I (defined as the radial distance from the origin of coordinates) were obtained.
2. The residual data was obtained by subtracting the mean lens $\bar{I}$ (already calculated and saved as a vector from the training set as indicated in ^16,17^), and projected into a basis of six *eigenlenses*:
$$\begin{array}{l} \;\;\;\;\;\;r=I-\bar{I},\\ a={\left(a_{1},\ldots ,a_{6}\right)}^{\text{T}}=M^{T}r={\left(e_{1}r,\ldots ,e_{6}r\right)}^{\text{T}}, \end{array}$$
  where a is a vector with the 6 coefficients, $a={\left(a_{1},\ldots ,a_{6}\right)}^{\text{T}}$ that represents the shape of the lens in an *eigenlens* basis; M is an M×6 matrix where each column is the ordered (starting from the highest eigenvalue) first 6 *eigenlenses* $M=\left(e_{1},\ldots ,e_{6}\right)$; and I is an M×1 vector of the data; T indicates the transpose operator, and bold indicates vectors and matrices. Six *eigenlenses* were chosen as they provided an optimal trade-off between accuracy and compactness ^16^.
3. Lens reconstruction: Once the vector of *eigenlenses* coefficients that defines the lens shape a have been obtained, the full shape $\hat{I}$ is estimated by projecting into the *eigenlenses* basis and adding the mean lens $\bar{I}$:
$$\hat{I}=\bar{I}+\sum_{k=1}^{K=6}a_{k}e_{k}.$$

This projection ensures that the final lens shape adheres to realistic anatomical variations observed in the training population, improving the robustness of the reconstruction. Figure 5B shows the *eigenlenses* projection (in black) superimposed to the data after the registration process.

### 2.4 Quantification

Once the 3-D models were constructed, various geometrical parameters from the crystalline lens full shape were quantified, specifically: (1) lens equatorial diameter (DIA); (2) lens surface area (LSA); and (3) lens volume (VOL). The LSA was estimated as the sum of the triangles formed by Delaunay triangulation; the VOL was estimated, using double integration over the region where the lens is defined, as the sum of the VOL of the anterior and the posterior parts of the lens ^17,18^.

### 2.5 Data analysis.

The normality of the data was assessed using the Shapiro–Wilk test. Since lens volume did not meet the assumption of normality, non-parametric analyses were performed. Specifically, Spearman’s rank correlation coefficient was used to evaluate the association between the geometrical parameters of the crystalline lens and age, obtaining the Spearman correlation coefficient (ρ) and the p-value for testing the null hypothesis of no correlation (p). Bland Altman plots and linear regression analysis were used to assess agreement between the proposed method and previously reported crystalline lens full shape estimation methods from the pupil information ^16,17^. The coefficient of variation (CV) was used to evaluate repeatability. For all analyses, statistical significance was defined as a p-value lower than 0.05. Calculations were obtained using MATLAB software (MathWorks, Natick, MA, USA).

## 3. Results

### 3.1 OCT images showing the crystalline lens periphery and full lens shape reconstruction

**Figure 6.**
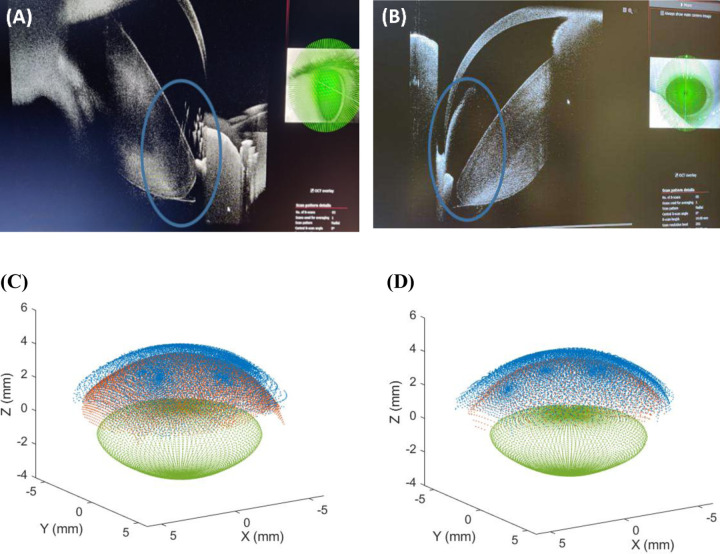
OCT raw images captured using lateral incidence (upper panels) and reconstructed 3−D geometry (lower panels). Blue ellipses in upper panels highlight the periphery of the crystalline lens, visible only under lateral incidence. (A) Subject #1, age 40 y/o, OS gazing temporally. (B) Subject #6, 26 y/o, OS gazing inferiorly. Surfaces in lower panels are represented by: blue for anterior surface; red for posterior surface; and green for the crystalline lens. (C) Subject #1. (D) Subject #6.

Figure 6 (upper panels) presents examples of raw OCT images of the crystalline lens periphery, acquired using the measurement protocol described above. Specifically, images are extracted from the volumetric measurements of two different subjects: (A) Subject #1, 40 y/o, OS gazing temporally; and (B) Subject #6, 26 y/o, OS gazing inferiorly. Figure 6 (lower panels) shows the corresponding full 3-D reconstruction for the same subjects.

**Figure 7.**
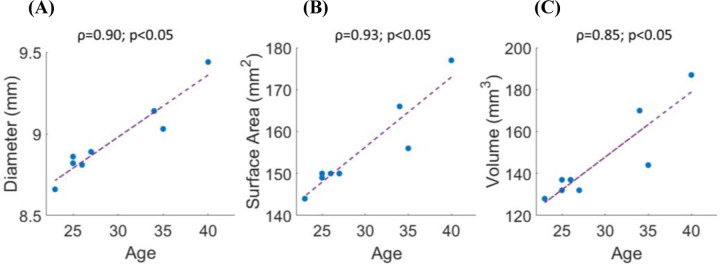
Correlation between the three quantified parameters and age. (A) Diameter (DIA); (B) Lens Surface Area (LSA); (C) Volume (VOL). Spearman correlation coefficient (ρ), p−values for testing the null hypothesis of no correlation (p), and best linear regression lines (purple dashed lines) are shown.

Figure7 shows the correlation between the quantified geometrical parameters and age. The Spearman correlation coefficient (ρ), p-values (p), and the best linear regression lines (purple dashed lines) are shown for each parameter. As age increased, each of the studied features showed a corresponding increase, indicating a trend of global morphological expansion. The correlation was high (ρ≥0.85) for all the parameters.

### 3.2 Repeatability

Three repeated measurements were obtained for two subjects (Subject # 1 and Subject # 3) to assess measurement repeatability. For subject # 1, the DIA values were 9.45 mm, 9.48 mm and 9.55 mm; the LSA values were 178 mm^2^, 179 mm^2^ and 181 mm^2^; and the VOL values were 188 mm^3^, 190 mm^3^ and 191 mm^3^. For subject # 3, the DIA values were 8.93 mm, 8.88 mm and 8.94 mm; the LSA values were 152 mm^2^, 150 mm^2^ and 152 mm^2^; and the VOL values were 135 mm^3^, 134 mm^3^ and 136 mm^3^. The coefficients of variation (CV) for Subject #1 were 0.54 % for DIA, 0.85% for LSA and 0.80 % for VOL, while for Subject #3, the CV values were 0.36 % for DIA, 0.76 % for LSA, and 0.74 % for VOL. These low CV values (all below 1 %) demonstrate high repeatability and consistency across repeated measurements for both subjects.

### 3.3 Validation of estimation methods: comparison with full shape estimation from the pupil using eigenlenses

We compared the geometric parameters (volume, equatorial diameter and lens surface area) obtained with the proposed method using lateral light incidence to those derived from the *eigenlenses* ^16,17^ estimation method, which reconstruct the full lens shape from the limited central region visible through the pupil under normal light incidence (standard OCT measurements commonly used in clinical imaging). This comparison allows to evaluate the accuracy and reliability of estimation techniques that rely on partial data when the full shape lens is not available.

**Figure 8.**
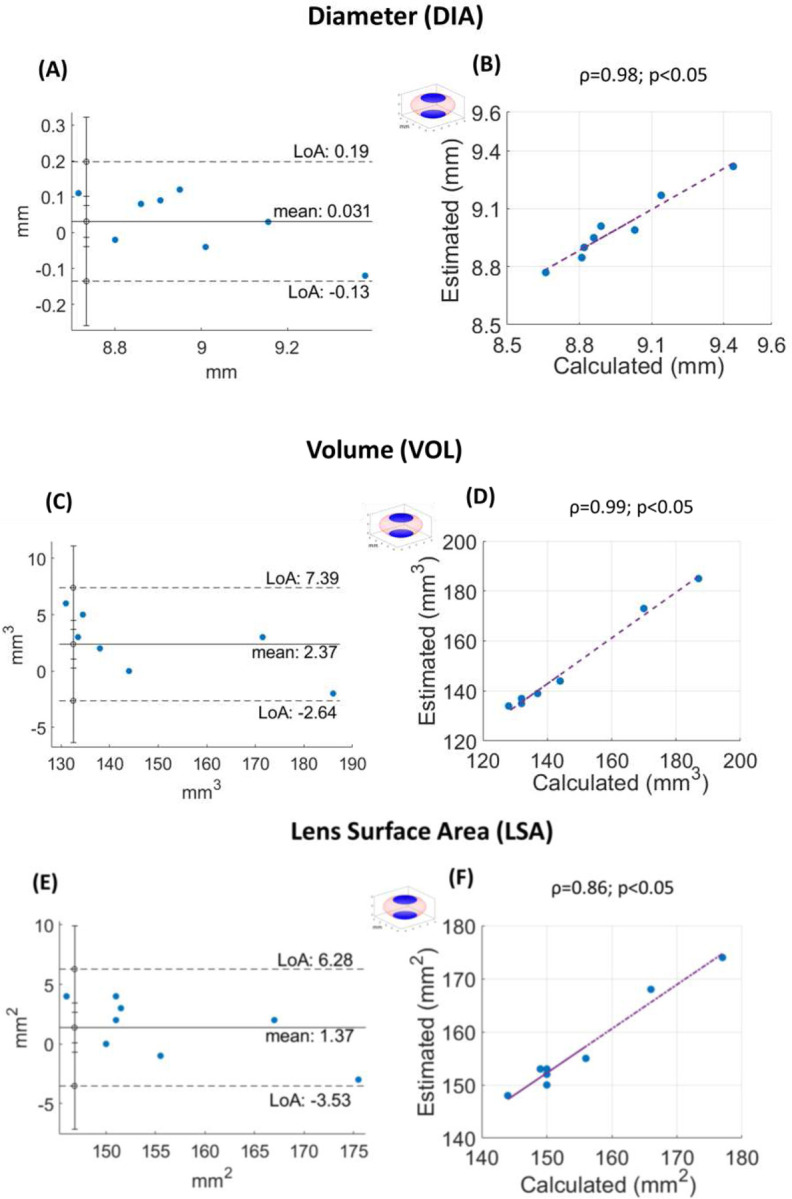
Comparison between the proposed method and estimation methods that estimate the full shape of the lens from the limited central zone (pupil) using *eigenlenses* ^16,17^. (A) Diameter, Bland−Altman plot; (B) Diameter, correlation plot; (C) Volume, Bland−Altman plot; (D) Volume, correlation plot; (E) Lens surface area, Bland−Altman plot; (F) Lens surface area, correlation plot. In the correlation plots, “Calculated” (X−axis) refers to the values obtained with the proposed method, and “estimated” (Y−axis) refers to the values obtained with estimation methods from the pupil. Spearman correlation coefficient (ρ), p−values for testing the null hypothesis of no correlation (p), and best linear regression lines (purple dashed lines) are shown.

Figure8 shows Bland-Altman (first column) and correlation (second column) plots por the lens DIA (first row) and VOL (second row). In Bland-Altman plots, the Y axis represents the mean difference (MD) between the methods calculated as estimation_method-proposed. The limits of agreement (LoA) are calculated as 1.96·SD, where SD is the standard deviation of the difference. In the correlation plots, the term “calculated” (X-axis) refers to the values obtained with the proposed method, and “estimated” (Y-axis) refers to the values obtained with estimation methods from the pupil (see the pupil blue area and the red color crystalline lens in the Y axis).

The Bland-Altman plots show a small MD and narrow LoA for DIA (MD=0.031 mm; LoA: −0.13 to 0.19 mm), VOL (MD=2.37 mm^3^; LoA: −2.64 to 7.39 mm^3^) and LSA (MD=1.37 mm^2^; LoA: −3.53 to 6.28 mm^2^). The correlation plot shows a high correlation between estimated and calculated values for DIA (ρ=0.98), VOL (ρ=0.99) and LSA (ρ=0.86). These results suggest strong agreement between both methods, demonstrating that *eigenlenses* full-shape estimation from the pupil ^16,17^ provides an accurate and consistent approximation of lens shape.

## 4. Discussion

Most in vivo studies of the crystalline lens have relied on optical imaging techniques restricted to the central region visible through the pupil. In this study, we report the ability to visualize the equatorial region of the crystalline lens in vivo using OCT, achieved with mydriasis and an off-axis (up to 45°) beam incidence. The equatorial region was successfully imaged in all eight subjects. Furthermore, we propose a method to merge on-axis and off-axis volumetric images to reconstruct the complete geometry of the crystalline lens.

Previous in vivo attempts to visualize the crystalline lens off-axis achieved a 17% increase in the visible areas of the lens ^21^, by combining on-axis OCT images with four off-axis volumes. In the present work, we employed eight volumes acquired at large angular incidences, facilitated by an external stimulus, enabling direct visualization of the lens equator for the first time (Figure 6). In addition, in contrast to ^21^, we reconstructed the three-dimensional geometry for the different incidence angles and registered the entire data, providing complete sampling of the posterior surface and, consequently, the full 3-D geometry of the crystalline lens.

In earlier works ^15–17^, we estimated the full shape of the crystalline lens from the regions of the anterior and posterior surfaces visible through the pupil. Using information of the full geometry of the crystalline lens that is accessible ex vivo, we developed mathematical models, so-called *eigenlenses*, to estimate the complete lens geometry based only on the visible portion. In the current work, by contrast, these parameters can be directly measured thanks to visualization of the entire crystalline lens. Although in some cases the full equator is not visible in all off-axis views, or the anterior corneal surface is partially obscured, preventing optical distortion correction of the subsequent surfaces and thus the use of that information in the reconstruction, the missing data are completed using *eigenlens* projections. Importantly, this does not represent an extrapolation, as in our earlier estimation-based approaches, but rather an interpolation of small unrecovered portions. This distinction is clearly illustrated in Supplementary Fig. S5, which compares the geometry accessible through the pupil with that obtained using the proposed method.

Repeatability analysis revealed low coefficients of variation for all geometrical parameters in two subjects (CV < 1%), demonstrating excellent consistency across repeated measurements. The proposed method has been used as the ground truth to evaluate the accuracy of estimation approaches ^16,17^. Specifically, Bland-Altman and correlation plots have been used to compare the lens diameter and volume obtained with the proposed and with estimation methods (Figure8). Overall, the results demonstrate that the *eigenlens* estimation method provides an accurate approximation of the full crystalline lens parameters, making it a valuable alternative when peripheral visualization is not available and in clinical settings where rapid measurements are required.

A strong positive correlation with age was observed for all three quantified parameters, indicating age-related lens growth: diameter (ρ = 0.90), volume (ρ = 0.85), and surface area (ρ = 0.93). Although this was not the primary objective of the study and the sample size was limited (n = 8), the observed trends are consistent with previous findings obtained through estimation methods and other imaging techniques ^37^.

It is important to perform optical distortion correction using three-dimensional ray-tracing methods, rather than the two-dimensional corrections typically implemented in commercial systems. Under on axis light incidence during meridional scanning, the ray is refracted within the same plane as the OCT meridian, making 2-D correction sufficient. However, in cases of lateral light incidence, as in the present work, the ray is refracted outside the OCT meridian plane. In such conditions, 3-D correction is essential to achieve accurate results.

A potential source of error could be the lack of precision in the incidence angles for each off-axis measurement. However, acquiring images at various incidence angles provided some redundancy (i.e., information present in multiple angular measurements), alleviating the need to know the exact off-axis angle a priori (in addition, it can be determined a posteriori from the data). Nevertheless, once registered, this redundancy proved useful for verifying the accuracy of the registration process. The number and distribution of incidence angles could be further optimized to reduce acquisition time in clinical applications.

The gradient index of refraction (GRIN) within the crystalline lens affects the reconstruction of the posterior lens surface. However, the in vivo GRIN is not fully known. A limitation of the current study is that we assumed a constant refractive index of n = 1.417 for the crystalline lens. To assess the impact of this assumption, we reconstructed the posterior surface using three different refractive indices for the crystalline lens (n = 1.40, 1.417, and 1.44) for Subject # 6, covering the range of feasible values reported in the literature ^32,33^. The DIA varied by approximately 0.4% (8.90 mm, 8.90 mm, and 8.86 mm, respectively), the VOL by 3% (142 mm^3^, 140 mm^3^, and 137 mm^3^), and the LSA by 2% (142 mm^3^, 140 mm^3^, and 137 mm^3^) across refractive indices. Thus, the influence of the refractive index can be considered negligible. Additionally, in some cases, the iris was not clearly visible in some OCT images at very high incidence angles. Nevertheless, this affected less than 5 % of the meridians and therefore did not impact the estimation of the iris plane and center, which are required for the registration process.

## 5. Conclusion

In this study, three-dimensional reconstruction of the full crystalline lens shape from OCT images was achieved using lateral light incidence and a methodology to merge all measurements from the different angles. The results showed that quantified parameters of the crystalline lens (diameter, surface area, and volume) increased with age. Furthermore, the study demonstrated that the full lens shape estimated from the pupil region using *eigenlenses* closely matched the geometry obtained by registering multiple OCT views at different incidences. This agreement underscores the potential of eigenlens-based approaches to accurately reconstruct the full lens shape in clinical settings.

Enhanced visibility of the lens periphery, combined with the ability to fully quantify its geometry, may improve understanding and assessment of lens accommodation, contribute to studies of myopia progression and presbyopia onset, and support the design and customization of intraocular lens (IOL) implants, ultimately enhancing visual outcomes and patient satisfaction in cataract and refractive surgeries.

## Supplementary Material

Supplement 1

**Supplementary information** accompanies this paper at.

## Data Availability

The datasets analysed during the current study are available in a github repository: EduardoMartinezEnriquez/Complete−reconstruction−crystalline−lens−shape: Data used in the paper “Complete reconstruction of the crystalline lens shape from OCT images acquired with off−axis viewing”. Data in Excel and Matlab formats (.mat).

## References

[1] Marcos S. Simulating Outcomes of Cataract Surgery: Important Advances in Ophthalmology. ARBE 277 (2021) doi:10.1146/annurev-bioeng-082420. 33848431

[2] Mutti D. 0 Optical and Structural Development of the Crystalline Lens in Childhood. Invest Ophtalmol Vis Sci. 39(1), 120–133 (1998). 9430553

[3] Zadnik K., Mutti D. 0, Fusaro R. E. & Adams A. J. Longitudinal evidence of crystalline lens thinning. Invest Ophtalmol Vis Sci. 36(8), 1581–1587 (1995). 7601639

[4] de la Hoz A., Martinez-Enriquez E. & Marcos S. Estimation of Crystalline Lens Material Properties From Patient Accommodation Data and Finite Element Models. Invest Ophthalmol Vis Sci 64, (2023). 10.1167/iovs.64.11.31 PMC10461688 37639248

[5] Huang D. Optical Coherence Tomography HHS Public Access. Science (1979) 254, 1178–1181 (1991). 10.1126/science.1957169 PMC4638169 1957169

[6] Atchison D. A. Age-related changes in optical and biometric characteristics of emmetropic eyes. J Vis 8, (2008). 10.1167/8.4.29 18484868

[7] Kasthurirangan S., Markwell E. L., Atchison D. A. & Pope J. M. MRI study of the changes in crystalline lens shape with accommodation and aging in humans. J Vis 11, 19 (2011). 10.1167/11.3.19 21441300

[8] Sheppard A. L. Three-dimensional magnetic imaging of the phakic crystalline lens during accommodation. Invest Ophthalmol Vis Sci 52, 3699–3700 (2011). 21632703 10.1167/iovs.11-7385

[9] Ramasubramanian V. & Glasser A. Objective measurement of accommodative biometric changes using ultrasound biomicroscopy. J Cataract Refract Surg 41, 511–526 (2015). 25804579 10.1016/j.jcrs.2014.08.033 PMC4374129

[10] Hermans E. A. Constant volume of the human lens and decrease in surface area of the capsular bag during accommodation: An MRI and Scheimpflug study. Invest Ophthalmol Vis Sci 50, 281–289 (2009). 18676625 10.1167/iovs.08-2124

[11] Yoo Y. S. Use of the Crystalline Lens Equatorial Plane as a New Parameter for Predicting Postoperative Intraocular Lens Position. Am J Ophthalmol 198, 17–24 (2019). 30236773 10.1016/j.ajo.2018.09.005

[12] Yoo Y. S., Whang W. J., Kim H. S., Joo C. K. & Yoon G. Preoperative biometric measurements with anterior segment optical coherence tomography and prediction of postoperative intraocular lens position. Medicine (United States) 98, (2019). 10.1097/MD.0000000000018026 PMC6922509 31852065

[13] Waring G. O., Chang D. H., Rocha K. M., Gouvea L. & Penatti R. Correlation of Intraoperative Optical Coherence Tomography of Crystalline Lens Diameter, Thickness, and Volume with Biometry and Age. Am J Ophthalmol 225, 147–156 (2021). 33385370 10.1016/j.ajo.2020.12.021

[14] Satou T. Relationship between Crystalline Lens Thickness and Shape and the Identification of Anterior Ocular Segment Parameters for Predicting the Intraocular Lens Position after Cataract Surgery. Biomed Res Int 2019, 1–9 (2019). 10.1155/2019/3458548 PMC6644274 31360711

[15] Martinez-Enriquez E. Optical coherence tomography based estimates of crystalline lens volume, equatorial diameter, and plane position. Invest Ophthalmol Vis Sci 57, OCT600–OCT610 (2016). 27627188 10.1167/iovs.15-18933

[16] Martinez-Enriquez E., de Castro A. & Marcos S. Eigenlenses: a new model for full crystalline lens shape representation and its applications. Biomed Opt Express 11, 5633–5649 (2020). 33149976 10.1364/BOE.397695 PMC7587276

[17] Martínez-Enríquez E. Estimation of the full shape of the crystalline lens in-vivo from OCT images using eigenlenses. Biomed Opt Express 14, 608–626 (2023). 36874490 10.1364/BOE.477557 PMC9979676

[18] Martinez-Enriquez E., Perez-Merino P., Velasco-Ocana M. & Marcos. Susana. OCT-based full crystalline lens shape change during accommodation in vivo. Biomed Opt Express 8, 918–933 (2017). 28270993 10.1364/BOE.8.000918 PMC5330589

[19] Muralidharan G. Morphological changes of human crystalline lens in myopia. Biomed Opt Express 10, 6084–6095 (2019). 31853387 10.1364/BOE.10.006084 PMC6913406

[20] Martínez-Enríquez E. Estimation of the full shape of the crystalline lens from OCT: validation using stretched donor lenses. Biomed Opt Express 14, 4261–4276 (2023). 37799671 10.1364/BOE.493795 PMC10549758

[21] Chen Y. Increased crystalline lens coverage in optical coherence tomography with oblique scanning and volume stitching. Biomed Opt Express 12, 1529–1542 (2021). 33796370 10.1364/BOE.418051 PMC7984769

[22] Laves M. H., Kahrs L. A. & Ortmaier T. Volumetric 3D stitching of optical coherence tomography volumes. Current Directions in Biomedical Engineering 4, 327–330 (2018).

[23] Gan Y., Yao W., Myers K. M. & Hendon C. P. An automated 3D registration method for optical coherence tomography volumes. in 2014 36th Annual International Conference of the IEEE Engineering in Medicine and Biology Society, EMBC 2014 3873–3876 (Institute of Electrical and Electronics Engineers Inc., 2014). doi:10.1109/EMBC.2014.6944469. PMC6080205 25570837

[24] Finke M. Automatic scanning of large tissue areas in neurosurgery using optical coherence tomography. International Journal of Medical Robotics and Computer Assisted Surgery 8, 327–336 (2012). 22911978 10.1002/rcs.1425

[25] Zawadzki R. J. Improved representation of retinal data acquired with volumetric Fd-OCT: co-registration, visualization, and reconstruction of a large field of view. Ophthalmic Technologies XVIII 6844, 40–47 (2008).

[26] Asam J. S., Polzer M., Tafreshi A., Hirnschall N. & Findl O. Anterior Segment OCT. in High Resolution Imaging in Microscopy and Ophthalmology 285–299 (Springer International Publishing, Cham, 2019). 32091839

[27] Pérez-Merino P., Velasco-Ocana M., Martinez-Enriquez E. & Marcos S. OCT-based crystalline lens topography in accommodating eyes. Biomed Opt Express 6, 5039–5054 (2015). 26713216 10.1364/BOE.6.005039 PMC4679276

[28] Zernike F. Beugungstheorie des schneidenverfahrens und seiner verbesserten form, der phasenkontrastmethode. Physica 1, 689–704 (1934).

[29] Wyant J. C. & Creath K. Basic Wavefront Aberration Theory for Optical Metrology. in Applied Optics and Optical Engineering (eds. Shannon R. & Wyant J.) vol. 11 28–39 (Academic Press, New York, 1992).

[30] Ortiz S. Optical distortion correction in Optical Coherence Tomography for quantitative ocular anterior segment by three-dimensional imaging. Biomed Opt Express 18, 2782–2796 (2010). 10.1364/OE.18.002782 20174107

[31] Drexler W. Submicrometer Precision Biometry of the Anterior Segment of the Human Eye. Invest Ophtalmol Vis Sci. 38, 1304–1313 (1997). 9191593

[32] Borja D. Distortions of the posterior surface in optical coherence tomography images of the isolated crystalline lens: effect of the lens index gradient. Biomed Opt Express 1, 1331–1340 (2010). 21258553 10.1364/BOE.1.001331 PMC3018115

[33] Natarajan R. Age dependence of the average refractive index of the isolated human crystalline lens. Biomed Opt Express 15, 5901–5911 (2024). 39421764 10.1364/BOE.536501 PMC11482174

[34] Paul J. B., IEE, member & Neil, D. M. A method for registration of 3-D shapes. Transaction on pattern analysis and machine intelligence 14, 239–256 (1992).

[35] Martinez-Enriquez E., P. Pérez-Merino, Durán-Poveda S., I. Jiménez-Alfaro & Marcos S. Estimation of intraocular lens position from full crystalline lens geometry: Towards a new generation of intraocular lens power calculation formulas. Sci Rep 8, (2018). 10.1038/s41598-018-28272-6 PMC6026180 29959385

[36] Martinez-Enriquez E. Postoperative intraocular lens tilt from preoperative full crystalline lens geometry using machine learning. Biomed Opt Express 16, 1439–1456 (2025). 10.1364/BOE.551733 PMC12047715 40321992

[37] Martinez-Enriquez E., de Castro, A., Ruggeri, M., Manns, F. & Marcos, S. Crystalline Lens Optics. in Encyclopedia of the Eye (Second Edition) (ed. D’Amore P. A.) 76–89 (Elsevier, 2025).

