# Supplementary material for: Complete reconstruction of the crystalline lens shape from OCT images acquired with off-axis viewing
Source: Res Sq. 2025 Dec 4:rs.3.rs-7957290. Preprint. [Version 1] doi: 10.21203/rs.3.rs-7957290/v1 (PMC12687823; doi:10.21203/rs.3.rs-7957290/v1)
Supplement: Supplement 1 [file NIHPPrs7957290v1-supplement-1.pdf]

Supplementary Files

This is a list of supplementary files associated with this preprint. Click to download.

- SupplementaryFigureS1.png
- SupplementaryFigureS2.png
- SupplementaryVideoS3.mov
- SupplementaryVideoS4.mov
- SupplementaryFigureS5.png
- CaptionsSupplementary.docx
